# Management of Multiple Mandibular Gingival Recessions Following Orthodontic Treatment Using a Connective Tissue Graft and a Coronally Advanced Flap: A Case Report

**DOI:** 10.7759/cureus.113687

**Published:** 2026-07-30

**Authors:** Keily J Portillo Avila, Claudia M Ramirez, Angel R Esquea Rosa, Arianne C Acosta Sumoza, Maria D Moreno

**Affiliations:** 1 Dentistry, Dental Loft, San Pedro Sula, HND; 2 Dentistry, Overseas Dental Specialty Center, Key Largo, USA; 3 Dentistry, Private Practice, Thonotosassa, USA; 4 Dentistry, Kissimmee Oral Surgery, Kissimmee, USA; 5 Dentistry, Ventura Dental, Kissimmee, USA

**Keywords:** connective tissue graft, coronally advanced flap, gingival recession, mandibular anterior teeth, mucogingival surgery, orthodontic complications, periodontal esthetics, periodontal plastic surgery, root coverage, soft tissue grafting

## Abstract

Gingival recession is a common mucogingival condition that may develop following orthodontic treatment, particularly in patients with a thin periodontal phenotype and limited buccal bone support.

This case report describes the management of multiple mandibular anterior gingival recessions in a 29-year-old female patient affecting teeth #22 through #27. Clinical examination revealed extensive root exposure and reduced gingival tissue thickness in the mandibular anterior region, resulting in esthetic concerns and increased risk of root sensitivity. Cone beam computed tomography (CBCT) was utilized to evaluate the periodontal anatomy and assist in surgical planning. A coronally advanced flap combined with an autogenous connective tissue graft harvested from the palate was performed to achieve root coverage and increase soft tissue thickness. The palatal donor site was closed with sutures following graft harvesting, and the connective tissue graft was stabilized at the recipient site before coronal repositioning of the flap and final closure with non-resorbable sutures. Healing progressed uneventfully, with favorable tissue integration and significant improvement in gingival margin position observed at the two-week follow-up. One month after surgery, the patient demonstrated increased gingival thickness, substantial root coverage, improved periodontal health, and satisfactory esthetic outcomes.

This case highlights the effectiveness of a connective tissue graft combined with a coronally advanced flap for the treatment of orthodontically associated multiple gingival recessions and demonstrates predictable short-term clinical and esthetic results.

## Introduction

Gingival recession is characterized by the apical displacement of the gingival margin relative to the cementoenamel junction, resulting in exposure of the root surface. This condition is common in clinical practice and may lead to dentin hypersensitivity, root caries, plaque accumulation, cervical abrasion, and esthetic concerns, particularly when multiple anterior teeth are involved [1,2]. The etiology of gingival recession is multifactorial and includes traumatic toothbrushing, periodontal disease, anatomical factors such as a thin periodontal phenotype and alveolar bone dehiscence, as well as orthodontic tooth movement in susceptible patients [3-5].

Orthodontic treatment has been associated with the development or progression of gingival recession when excessive labial movement of teeth occurs beyond the alveolar housing or when treatment is performed in individuals with limited periodontal support [6,7]. Careful diagnosis of the periodontal phenotype and evaluation of the surrounding hard and soft tissues are essential to identify patients at increased risk and to facilitate appropriate treatment planning [8,9].

Among the available root coverage procedures, a coronally advanced flap combined with an autogenous connective tissue graft is considered the gold standard for the treatment of multiple gingival recessions because of its predictable root coverage, increased gingival thickness, improved long-term tissue stability, and favorable esthetic outcomes [10-17]. Cone beam computed tomography (CBCT) may also provide valuable information regarding the thickness of the facial alveolar bone and the presence of dehiscence or fenestration, thereby assisting in surgical planning.

This report describes the successful management of multiple mandibular anterior gingival recessions that developed following orthodontic treatment using a connective tissue graft in combination with a coronally advanced flap, resulting in favorable short-term clinical and esthetic outcomes.

## Case presentation

A 29-year-old healthy female presented for periodontal evaluation because of progressive gingival recession affecting the mandibular anterior teeth following completion of orthodontic treatment. Her medical history was noncontributory, and she reported no systemic diseases, smoking history, or use of medications known to affect periodontal health. The patient's primary concerns were the unaesthetic appearance of the exposed root surfaces and the potential for future dentin hypersensitivity.

**Figure 1:**
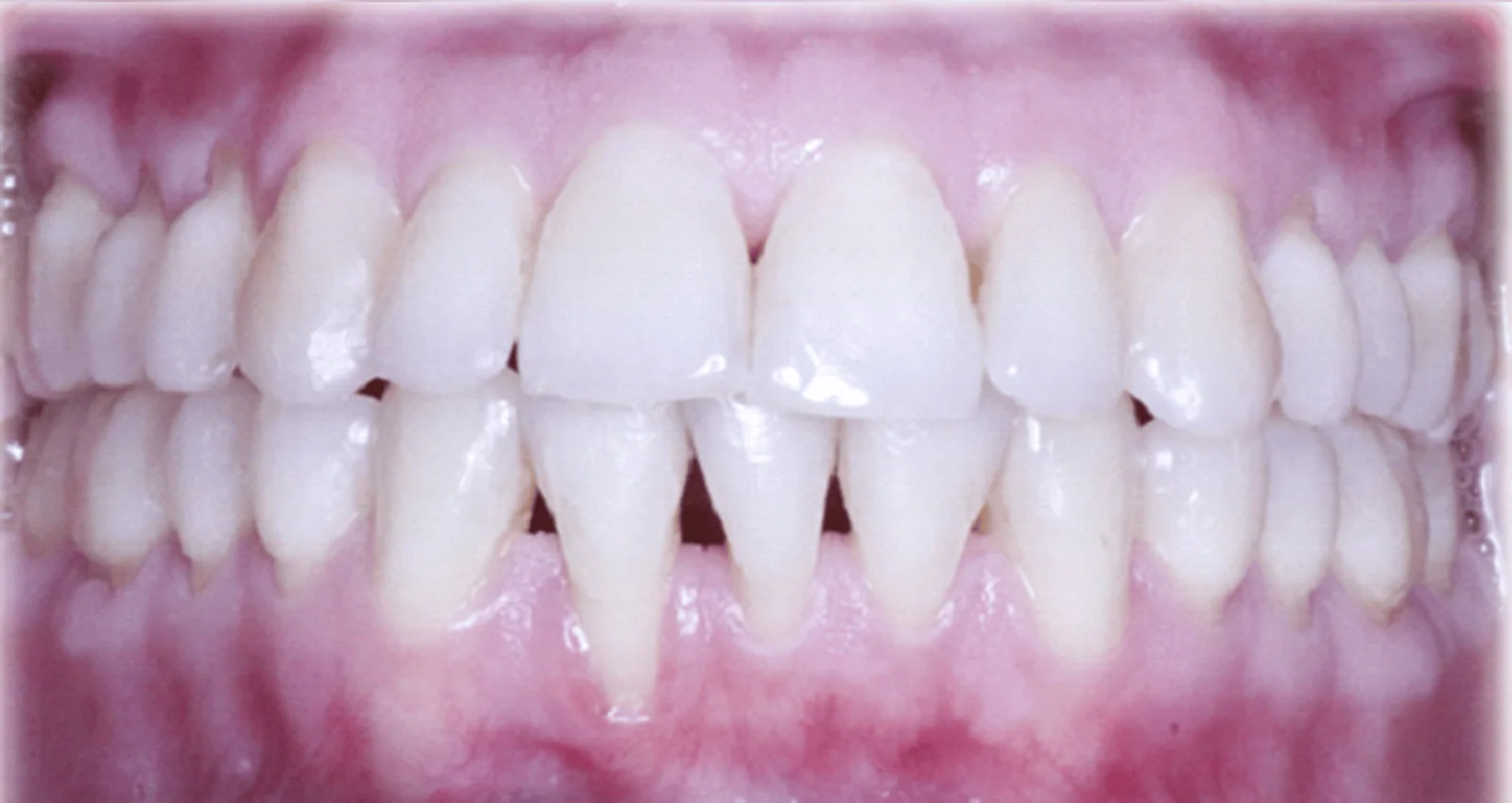
Preoperative clinical appearance following orthodontic treatment. Frontal intraoral photograph demonstrating multiple gingival recessions involving the mandibular anterior teeth (#22-#27) following completion of orthodontic treatment. Generalized root exposure and a thin periodontal phenotype are evident before surgical treatment with a connective tissue graft and a coronally advanced flap.

Clinical examination revealed multiple gingival recession defects involving teeth #22 through #27, with a thin periodontal phenotype and reduced width of keratinized tissue in the mandibular anterior region (Figure 1). The recession defects were associated with visible root exposure without evidence of active periodontal inflammation or increased probing depths. Plaque control was satisfactory, and the remaining dentition demonstrated stable periodontal support.

**Figure 2:**
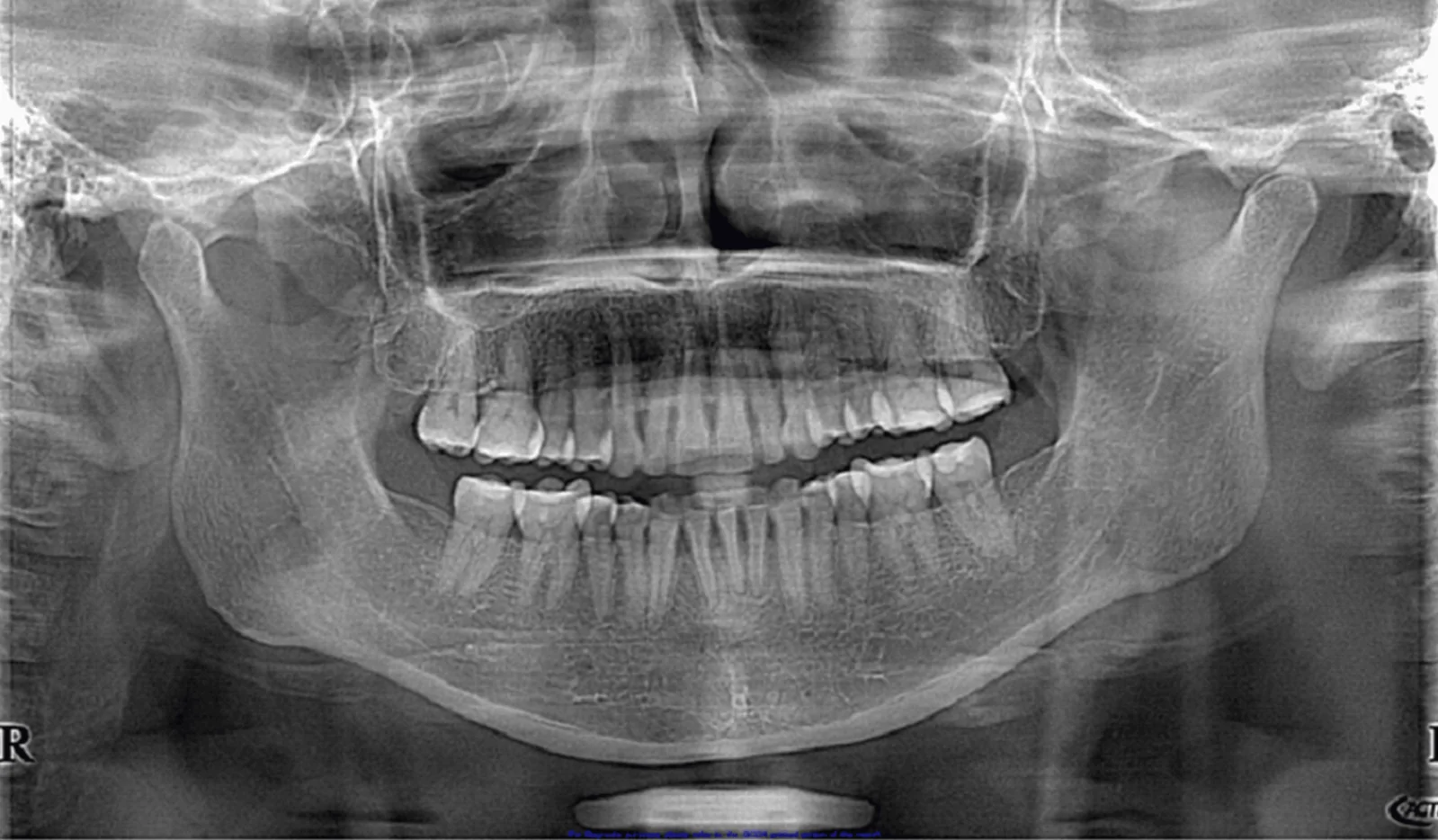
Preoperative panoramic radiograph. Preoperative panoramic radiograph obtained during the diagnostic evaluation. The image demonstrates generalized dentition with no significant periodontal bone loss or other radiographic pathology. The mandibular anterior teeth exhibit normal supporting alveolar bone levels, and the radiographic findings were used in conjunction with the clinical examination and cone-beam computed tomography for comprehensive treatment planning.

**Figure 3:**
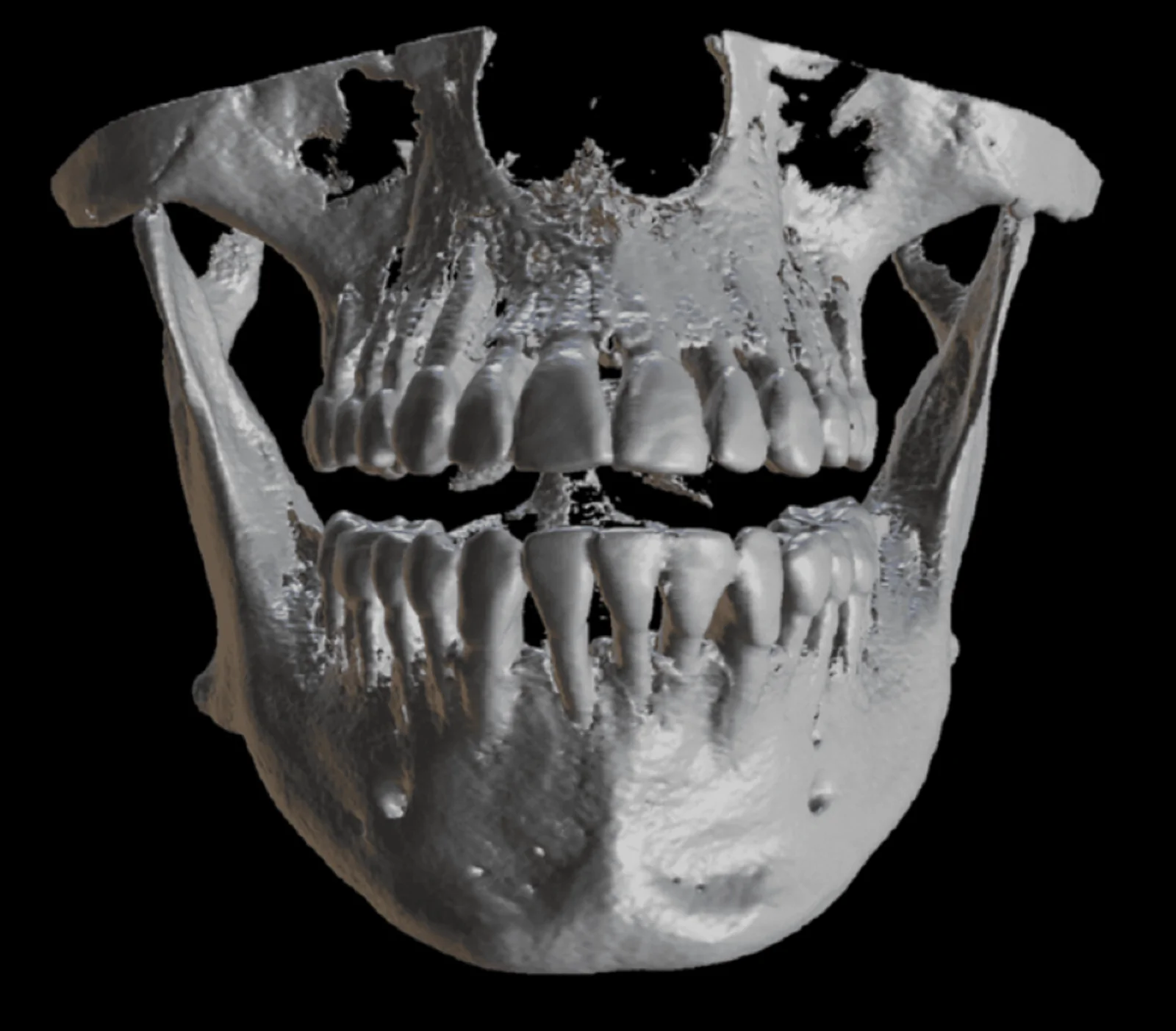
Three-dimensional CBCT reconstruction of the mandibular anterior region. Three-dimensional cone-beam computed tomography (CBCT) reconstruction demonstrating reduced facial alveolar bone support in the mandibular anterior region, with areas of facial alveolar bone loss (dehiscence) associated with the teeth affected by gingival recession. These findings supported the diagnosis and assisted in planning root coverage using a connective tissue graft and a coronally advanced flap.

A panoramic radiograph was obtained as part of the comprehensive evaluation and demonstrated no significant periodontal bone loss or other pathologic findings (Figure 2). Because panoramic radiography provides only a two-dimensional representation of the jaws and has limited sensitivity for detecting facial cortical bone defects, CBCT was performed for further evaluation. Three-dimensional CBCT reconstruction demonstrated a thin facial alveolar plate with localized facial alveolar dehiscence affecting the mandibular anterior teeth, findings that were not clearly evident on the panoramic radiograph (Figure 3). These findings confirmed the thin periodontal phenotype and supported the indication for periodontal plastic surgery.

Following clinical and radiographic evaluation, treatment options, risks, benefits, expected outcomes, and possible complications were discussed with the patient. Written informed consent was obtained before surgery.

**Figure 4:**
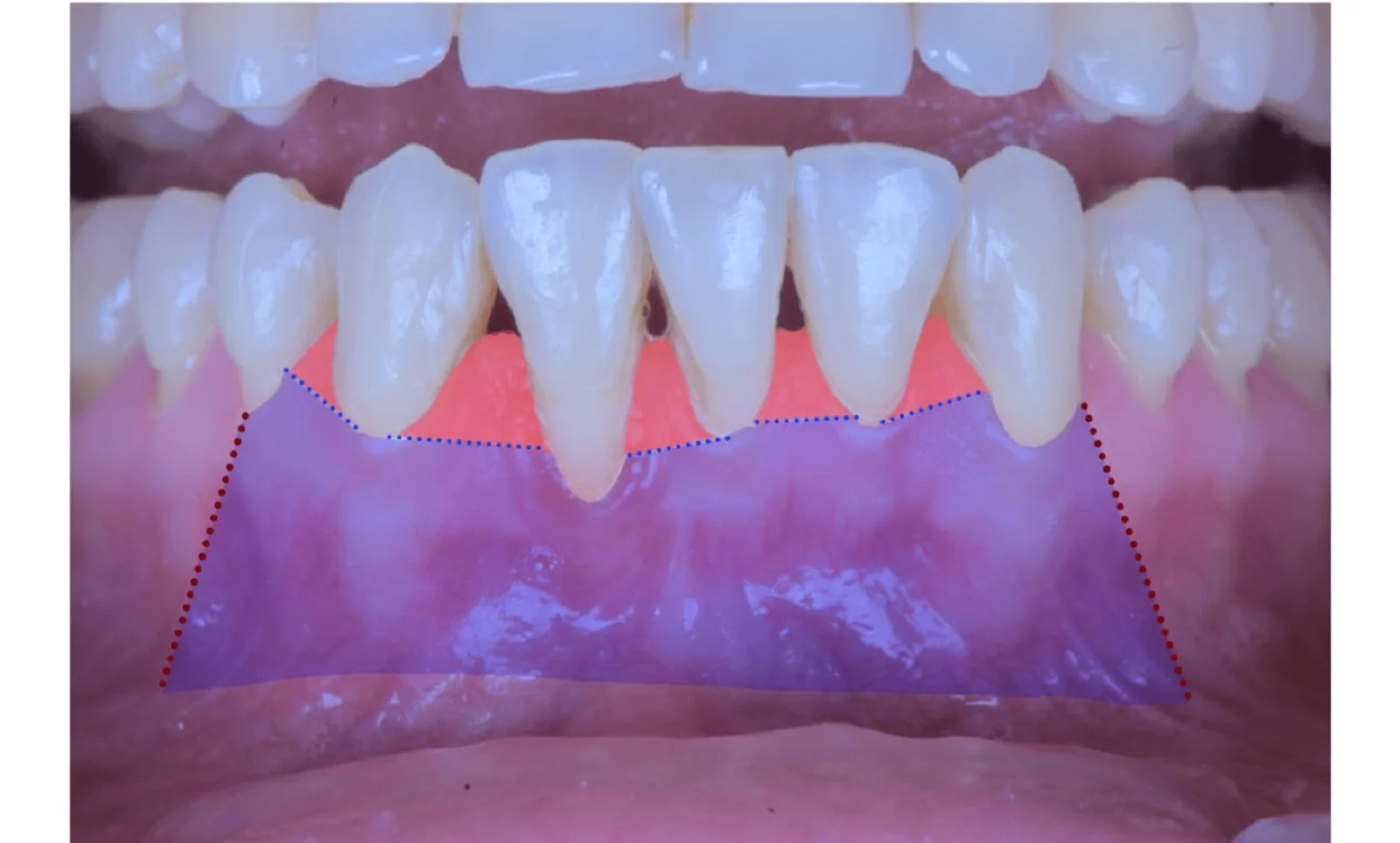
Surgical flap design based on CBCT evaluation. Preoperative design of the coronally advanced flap planned using the clinical examination and cone beam computed tomography (CBCT) findings. The incision outlines and flap extension were designed according to the areas of facial alveolar bone deficiency identified on CBCT to allow adequate flap mobilization, passive coronal advancement, and complete coverage of the connective tissue graft over the mandibular anterior gingival recessions.

**Figure 5:**
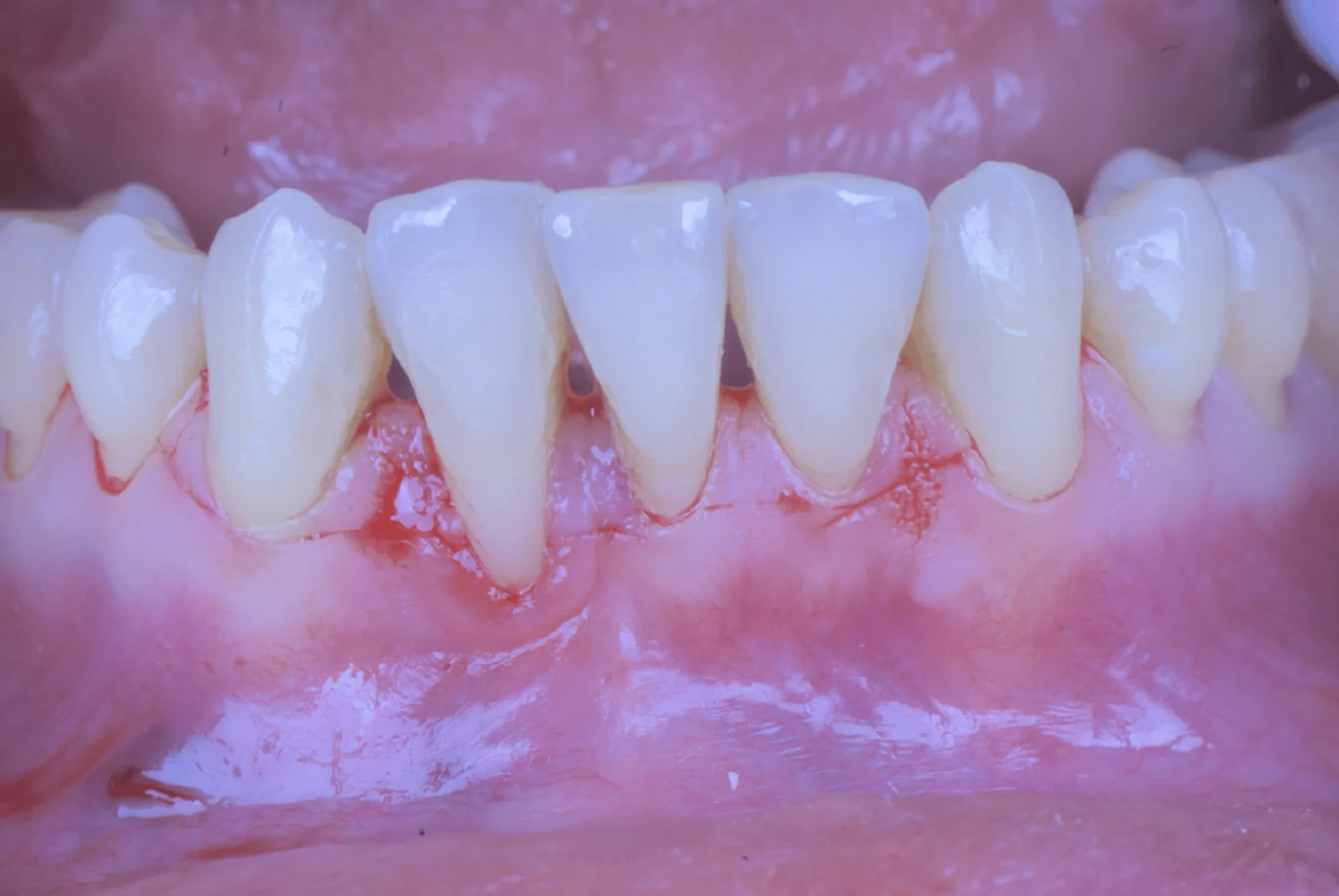
Initial flap incisions. Initial sulcular and releasing incisions were performed according to the preoperative flap design for the coronally advanced flap. The incisions were made to elevate the surgical flap while preserving the interdental papillae and ensuring adequate flap mobility for subsequent coronal advancement and placement of the connective tissue graft.

**Figure 6:**
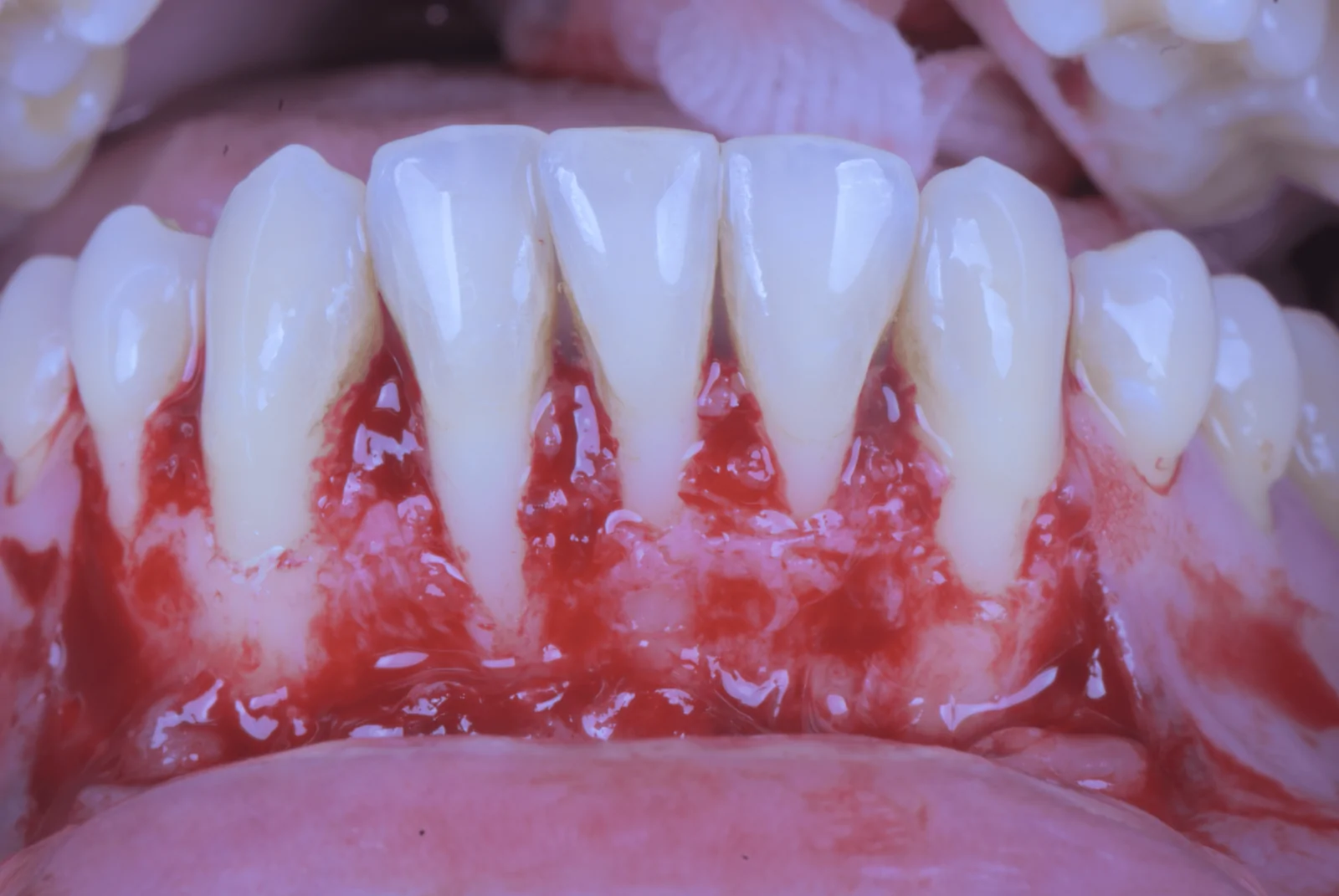
Recipient site preparation following flap elevation. Elevation of the coronally advanced flap exposing the recipient site in the mandibular anterior region. Following flap reflection, the exposed root surfaces were mechanically debrided, and the recipient bed was prepared to receive the autogenous connective tissue graft. Adequate flap mobility was achieved to permit passive coronal advancement and complete graft coverage.

After administration of local anesthesia, the surgical design for a coronally advanced flap was outlined according to the clinical examination and CBCT findings (Figure 4). Initial sulcular and releasing incisions were performed while preserving the interdental papillae (Figure 5). A split-full-thickness flap was carefully elevated to prepare the recipient site and allow passive coronal advancement while maintaining an adequate blood supply to the flap (Figure 6). The exposed root surfaces were thoroughly debrided and mechanically prepared before graft placement.

**Figure 7:**
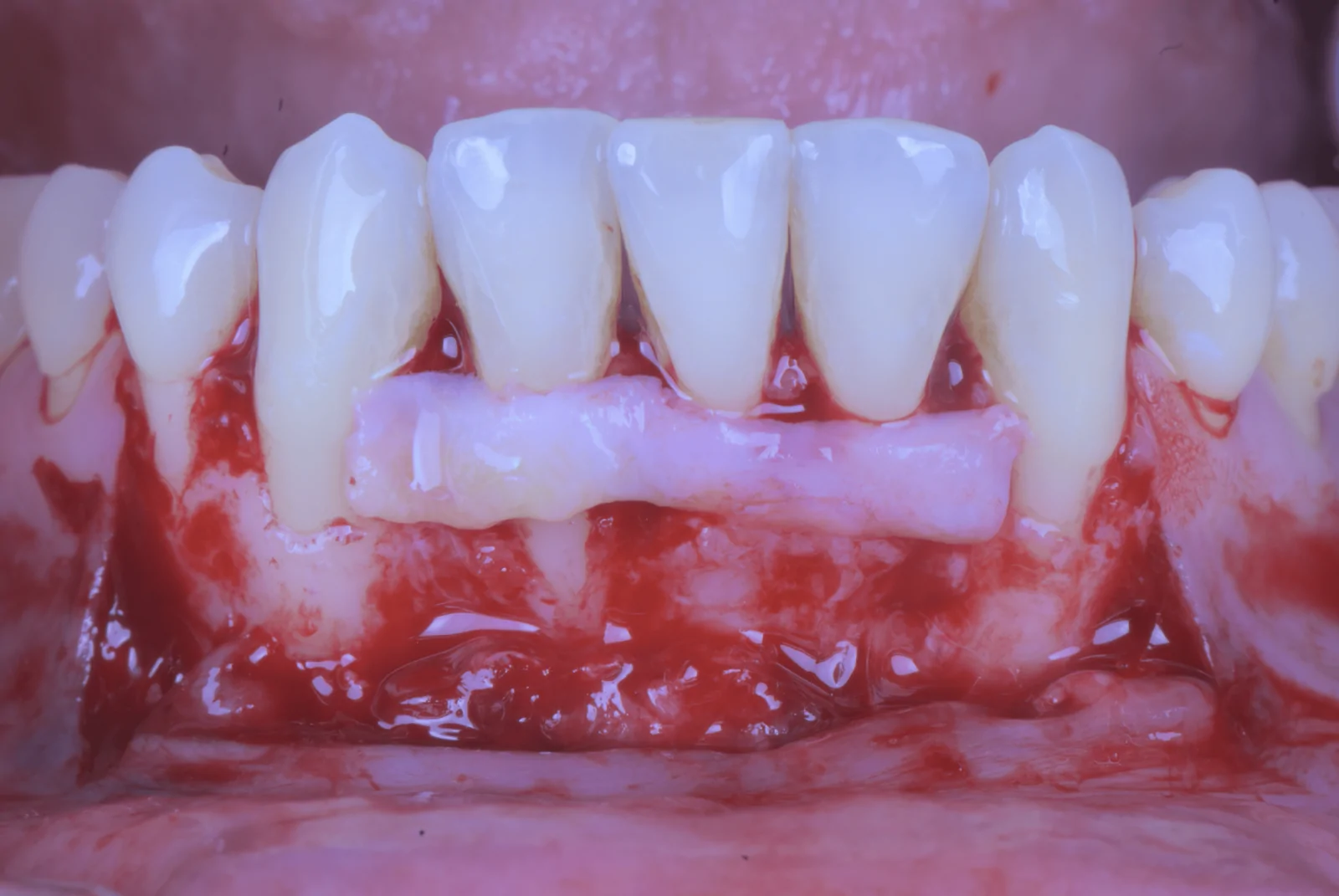
Placement and stabilization of the connective tissue graft. The autogenous connective tissue graft was positioned over the exposed root surfaces at the mandibular anterior recipient site and stabilized with interrupted sutures. The graft completely covered the recession defects, providing increased soft tissue thickness and a stable foundation for subsequent coronal advancement of the flap and root coverage.

**Figure 8:**
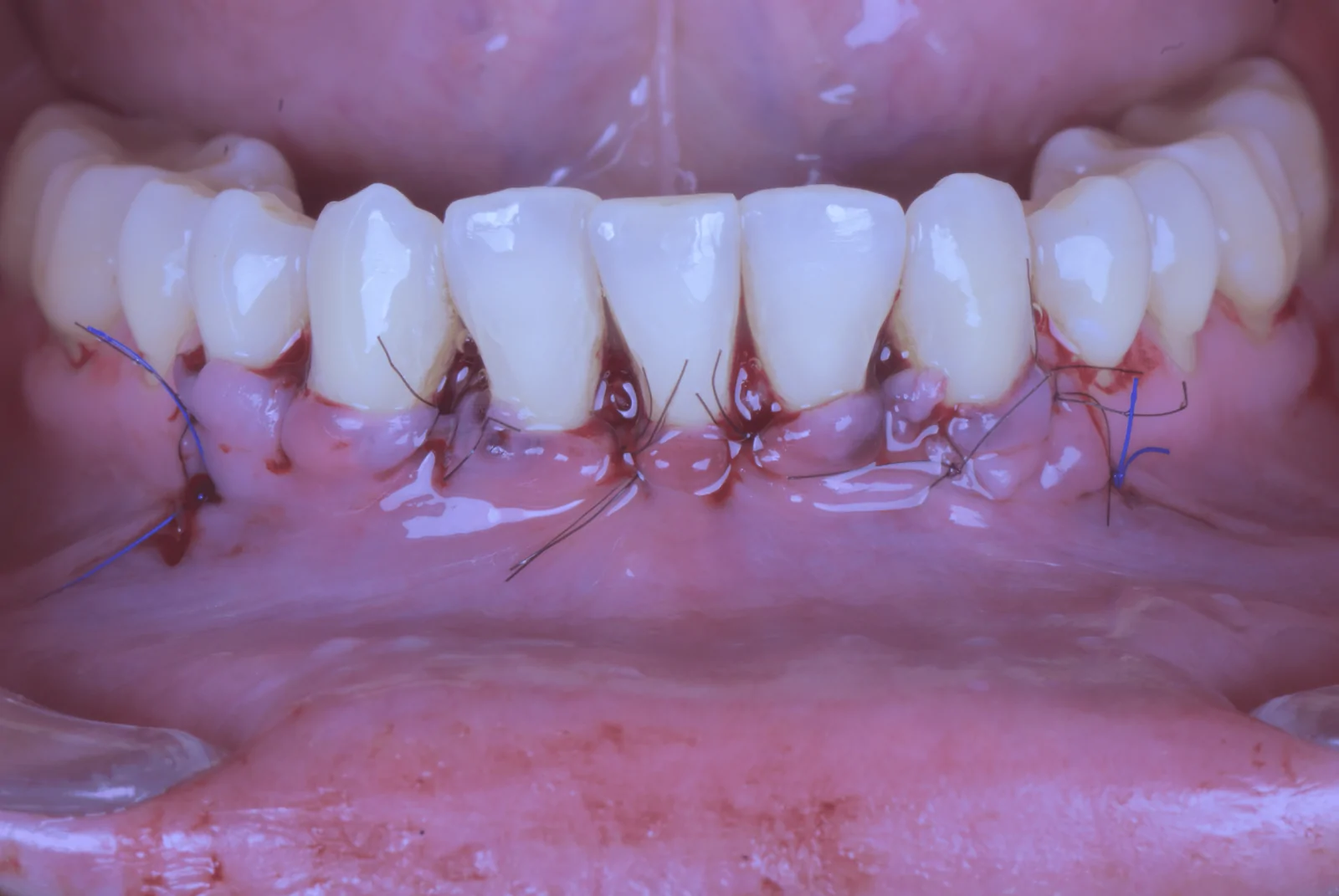
Coronally advanced flap with final suturing. The coronally advanced flap was passively repositioned to completely cover the connective tissue graft and secured with interrupted and sling sutures. Tension-free flap adaptation achieved complete coverage of the recession defects, promoting graft stability, optimal vascularization, and favorable conditions for root coverage and soft tissue healing.

**Figure 9:**
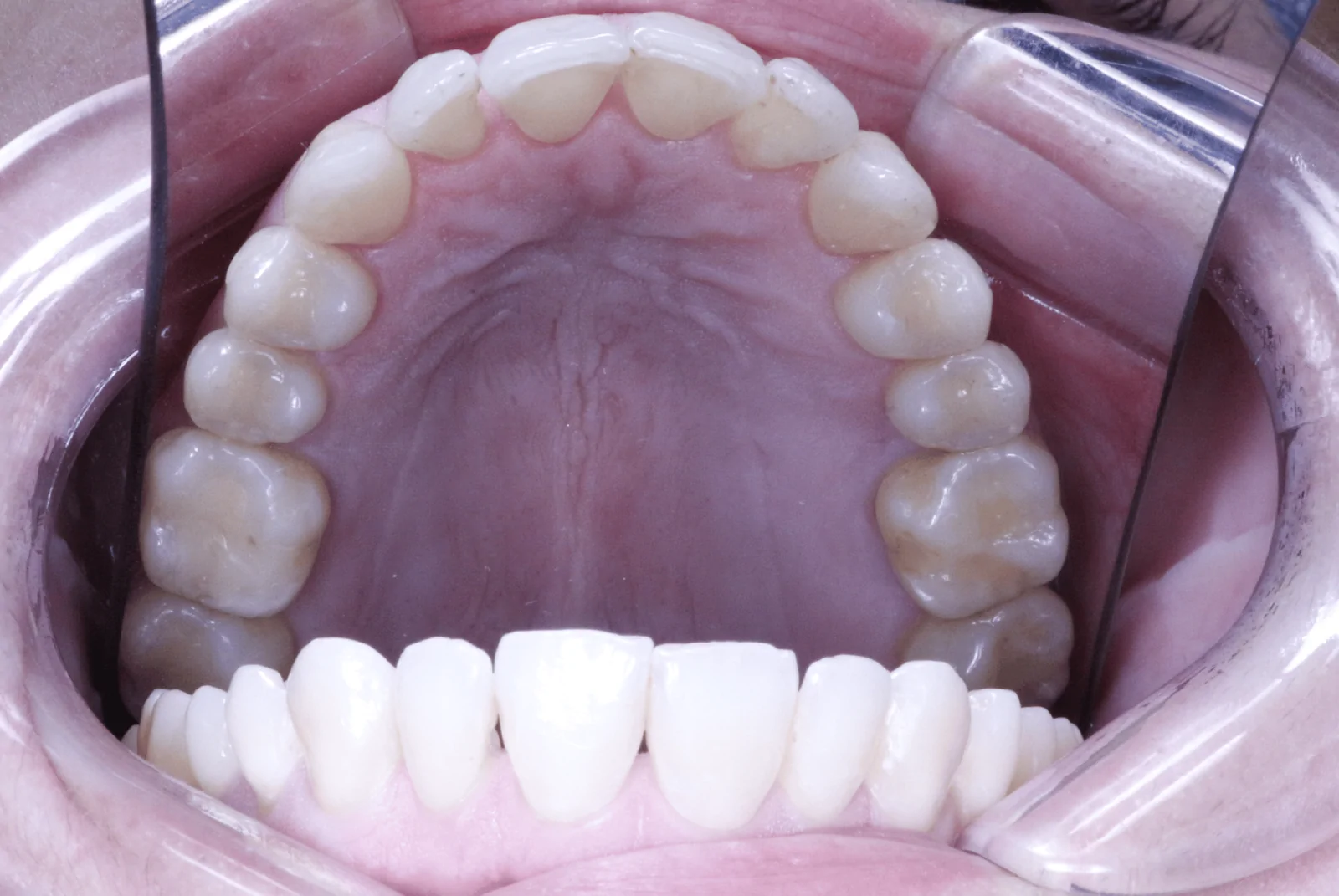
Preoperative palatal donor site. Preoperative occlusal view of the palate demonstrating the donor site selected for harvesting the autogenous connective tissue graft. The graft was obtained from the left posterior palatal mucosa and subsequently transferred to the mandibular anterior recipient site to increase soft tissue thickness and achieve root coverage.

The palatal donor site was identified and prepared before harvesting the graft (Figure 7). An autogenous subepithelial connective tissue graft was harvested from the left palate using the single-incision technique (Figure 8). The donor site was then closed with interrupted sutures to achieve primary wound closure, promote hemostasis, and facilitate uneventful healing (Figure 9).

**Figure 10:**
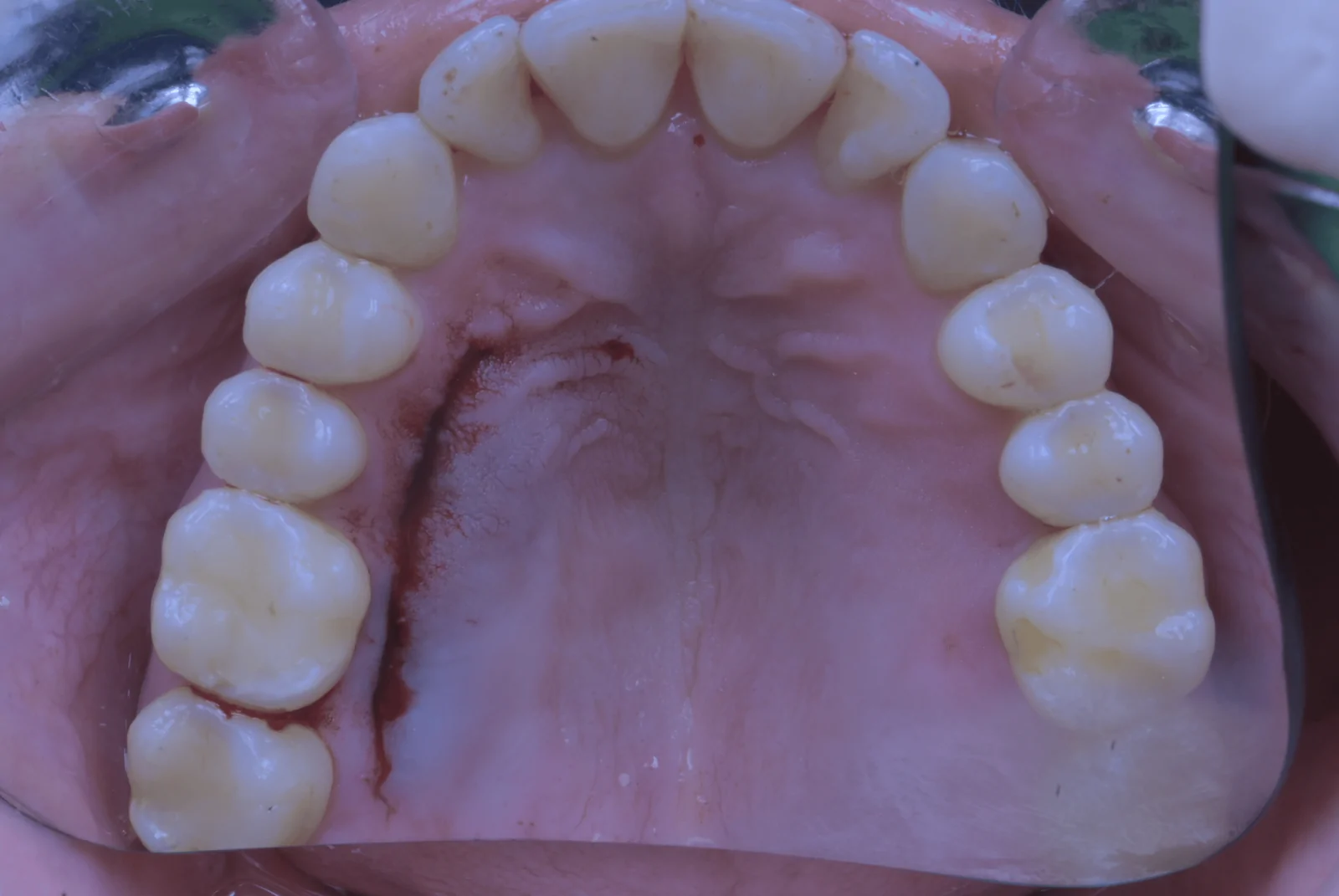
Palatal donor site after connective tissue graft harvesting. Occlusal view of the maxillary arch demonstrating the palatal donor site immediately after connective tissue graft harvesting. The connective tissue was obtained from the left palate, and the donor area was managed to achieve hemostasis while preserving the overlying palatal tissue, promoting uneventful healing and minimizing postoperative morbidity.

**Figure 11:**
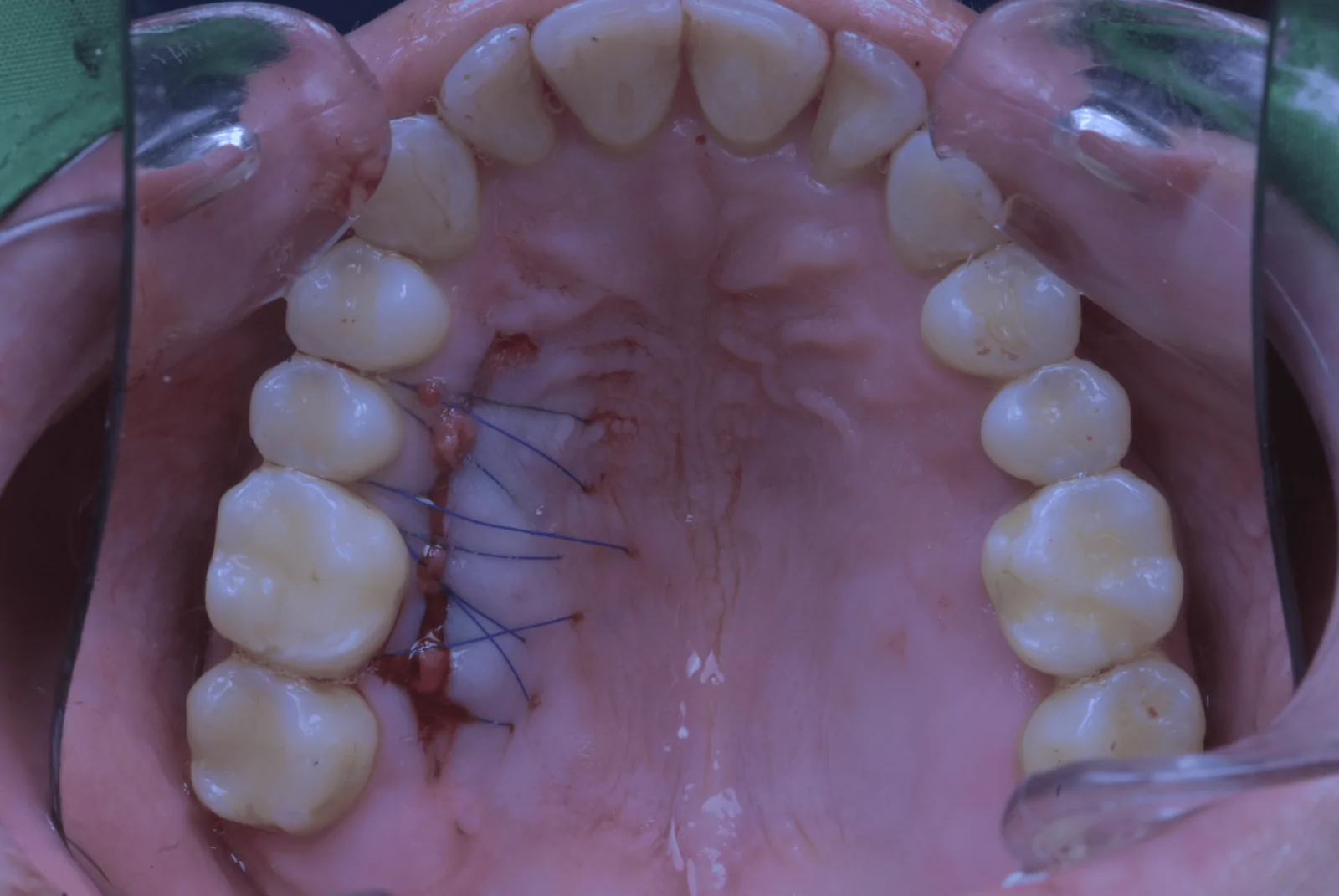
Primary closure of the palatal donor site after connective tissue graft harvesting. Occlusal view of the maxillary arch demonstrating primary closure of the palatal donor site following connective tissue graft harvesting. The donor area was sutured with interrupted sutures to achieve hemostasis, protect the surgical wound, reduce postoperative discomfort, and promote uneventful healing of the palate.

The harvested connective tissue graft was trimmed to the appropriate dimensions and stabilized over the prepared recipient bed using interrupted sutures, ensuring intimate adaptation to the exposed root surfaces and surrounding connective tissues (Figure 10). The recipient flap was subsequently advanced coronally to completely cover the graft and secured with suspended and interrupted nonresorbable sutures, achieving passive, tension-free adaptation and complete graft coverage (Figure 11). Adequate flap stability was confirmed before completion of the procedure.

The patient received standard postoperative instructions, including oral hygiene recommendations, twice-daily rinsing with 0.12% chlorhexidine gluconate for two weeks, analgesics as needed, and avoidance of mechanical trauma to the surgical site during the initial healing period.

Healing progressed uneventfully without postoperative complications. At the two-week follow-up appointment, the surgical site demonstrated satisfactory soft tissue healing with no evidence of infection, flap dehiscence, or graft necrosis. Sutures were removed, and oral hygiene instructions were reinforced.

**Figure 12:**
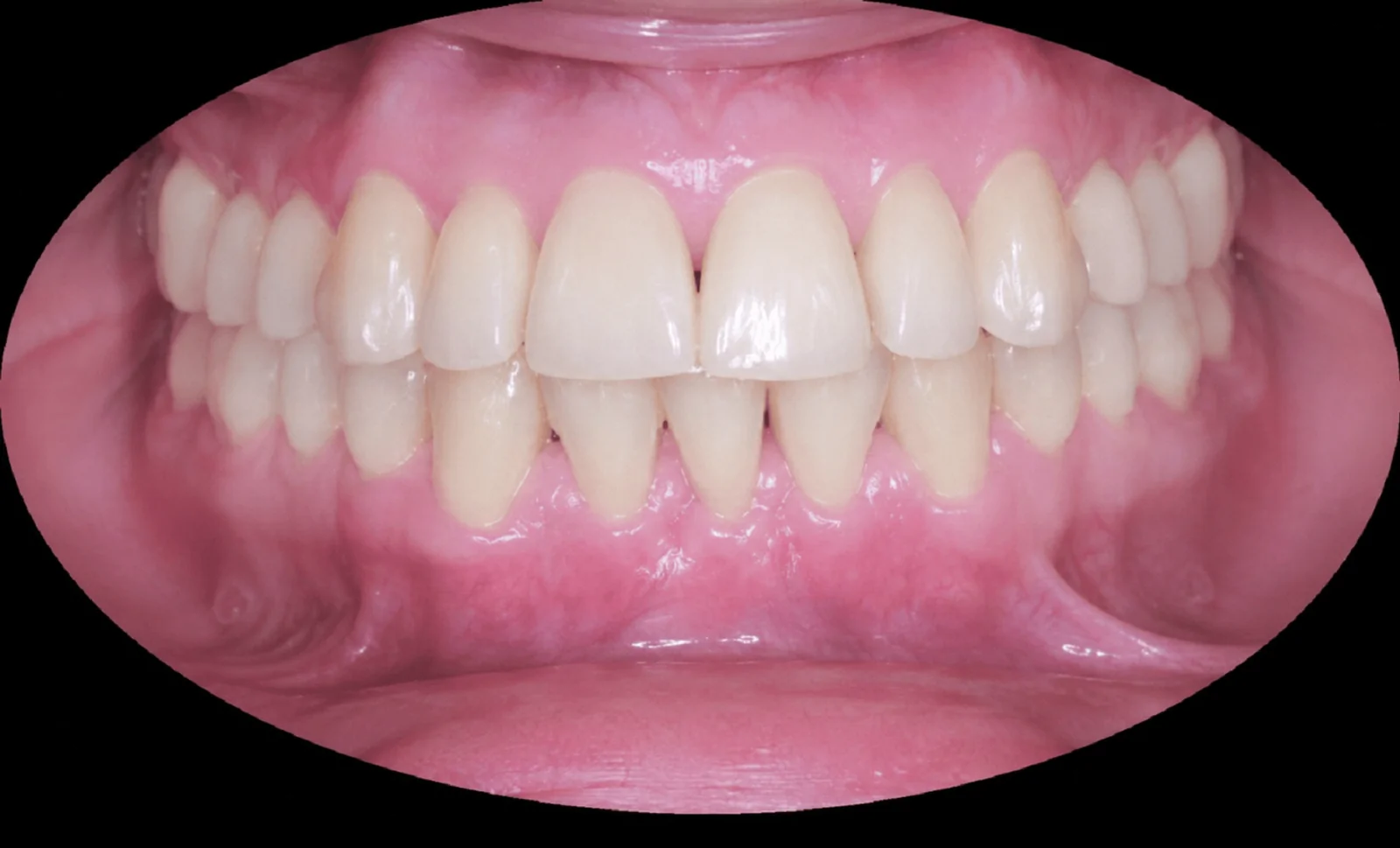
Six month postoperative outcome following coronally advanced flap and connective tissue graft. Frontal intraoral clinical photograph obtained at the postoperative follow-up demonstrating successful root coverage of the mandibular anterior teeth after treatment with a coronally advanced flap and subepithelial connective tissue graft. A significant increase in the width and thickness of the keratinized gingiva is evident, with harmonious gingival contours, improved soft tissue esthetics, and complete resolution of the previously exposed root surfaces. The treated tissues exhibit healthy color, firm consistency, and excellent integration with the adjacent gingiva, resulting in a stable and highly esthetic clinical outcome.

At the six-month postoperative evaluation, excellent root coverage was maintained with a marked increase in gingival thickness and width of keratinized tissue. The treated sites demonstrated healthy periodontal tissues, harmonious gingival contours, excellent integration of the connective tissue graft, and highly satisfactory esthetic outcomes (Figure 12).

## Discussion

Gingival recession is a common mucogingival condition characterized by apical migration of the gingival margin beyond the cementoenamel junction, resulting in root surface exposure [1,2]. Although the etiology is multifactorial, orthodontic treatment has been recognized as a potential contributing factor when tooth movement exceeds the limits of the alveolar housing, particularly in patients with a thin periodontal phenotype or preexisting facial alveolar bone dehiscence [3,4]. In susceptible individuals, labial movement of mandibular anterior teeth may reduce soft tissue support and increase the risk of recession development, emphasizing the importance of careful periodontal assessment before and during orthodontic treatment [6-9].

Successful management of multiple gingival recessions requires accurate diagnosis, appropriate case selection, and careful evaluation of both hard and soft tissue anatomy. In the present case, conventional panoramic radiography demonstrated no significant periodontal bone loss but was unable to adequately visualize the facial cortical plate. CBCT provided valuable three-dimensional information regarding the presence of facial alveolar dehiscence and reduced buccal bone thickness, findings that confirmed the patient's thin periodontal phenotype and facilitated surgical planning. Although CBCT is not routinely indicated for all mucogingival procedures, its selective use may improve diagnosis and treatment planning in complex cases where facial bone morphology cannot be adequately assessed using conventional imaging [8,10].

Among the various periodontal plastic surgical techniques available for root coverage, the combination of a coronally advanced flap with an autogenous subepithelial connective tissue graft remains the gold standard for the treatment of multiple adjacent gingival recessions [11,12]. This approach has consistently demonstrated high percentages of root coverage, increased gingival thickness, greater width of keratinized tissue, improved long-term tissue stability, and highly favorable esthetic outcomes [13,14]. The connective tissue graft enhances vascularization, increases soft tissue volume, and improves resistance to future recession, while the coronally advanced flap provides stable coverage of the graft and exposed root surfaces during healing [15-17].

In the present case, favorable clinical healing was observed without postoperative complications. At the six-month follow-up, substantial root coverage was maintained with increased gingival thickness, healthy periodontal tissues, harmonious gingival contours, and excellent esthetic integration. These findings are consistent with previous reports demonstrating predictable clinical outcomes following connective tissue grafting combined with coronally advanced flap procedures in patients with multiple gingival recessions.

Careful management of the palatal donor site also contributes to successful postoperative recovery. Primary closure of the donor site promotes hemostasis, minimizes postoperative discomfort, reduces the risk of bleeding, and facilitates uneventful healing. Appropriate flap design, passive coronal advancement, tension-free flap adaptation, meticulous suturing, and strict postoperative plaque control are additional factors that contribute to predictable wound healing and favorable surgical outcomes.

Despite the favorable clinical outcome, several limitations should be acknowledged. This report describes a single patient with a relatively short follow-up period, limiting the ability to generalize the results or evaluate long-term stability. Additional prospective studies with larger patient populations and extended follow-up are needed to further assess the long-term effectiveness of connective tissue grafting combined with coronally advanced flap procedures in patients with orthodontically associated gingival recession.

Overall, this case demonstrates that comprehensive periodontal evaluation, selective use of CBCT for diagnosis, and treatment with an autogenous connective tissue graft combined with a coronally advanced flap can provide predictable root coverage, increased soft tissue thickness, and excellent esthetic outcomes in appropriately selected patients presenting with multiple gingival recessions following orthodontic treatment.

## Conclusions

This case demonstrates that a coronally advanced flap combined with an autogenous connective tissue graft is an effective treatment for multiple mandibular gingival recessions associated with orthodontic therapy. Careful preoperative assessment, including selective use of CBCT when indicated, facilitated identification of the underlying periodontal anatomy and supported appropriate surgical planning. Favorable clinical healing, increased gingival thickness, substantial root coverage, and satisfactory esthetic outcomes were maintained at the six-month follow-up. Although longer-term studies involving larger patient populations are needed, this case supports the predictable use of connective tissue grafting with a coronally advanced flap for the management of orthodontically associated gingival recession in appropriately selected patients.
